# Balancing the excitability of M1 circuitry during movement observation without overt replication

**DOI:** 10.3389/fnbeh.2014.00316

**Published:** 2014-09-17

**Authors:** Pablo Arias, Verónica Robles-García, Yoanna Corral-Bergantiños, Nelson Espinosa, Laura Mordillo-Mateos, Kenneth Grieve, Antonio Oliviero, Javier Cudeiro

**Affiliations:** ^1^Laboratory of Neuroscience and Motor Control (NEUROcom), Department of Medicine-INEF-Galicia and Institute of Biomedical Research of Coruña (INIBIC), University of A CoruñaSpain; ^2^FENNSI Group, Hospital Nacional de Parapléjicos, Servicio de Salud de Castilla-La ManchaToledo, Spain; ^3^Faculty of Life Sciences, University of ManchesterManchester, UK

**Keywords:** movement observation, replica cancelation, motor cortex, human, mirror neuron system

## Abstract

Although observation of a movement increases the excitability of the motor system of the observer, it does not induce a motor replica. What is the mechanism for replica suppression? We performed a series of experiments, involving a total of 66 healthy humans, to explore the excitability of different M1 circuits and the spinal cord during observation of simple movements. Several strategies were used. In the first and second experimental blocks, we used several delay times from movement onset to evaluate the time-course modulation of the cortico-spinal excitability (CSE), and its potential dependency on the duration of the movement observed; in order to do this single pulse transcranial magnetic stimulation (TMS) over M1 was used. In subsequent experiments, at selected delay times from movement-onset, we probed the excitability of the cortico-spinal circuits using three different approaches: (i) electric cervicomedullary stimulation (CMS), to test spinal excitability, (ii) paired-pulse TMS over M1, to evaluate the cortical inhibitory-excitatory balance (short intracortical inhibition (SICI) and intracortical facilitation (ICF)], and (iii) continuous theta-burst stimulation (cTBS), to modulate the excitability of M1 cortical circuits. We observed a stereotyped response in the modulation of CSE. At 500 ms after movement-onset the ICF was increased; although the most clear-cut effect was a decrease of CSE. The compensatory mechanism was not explained by changes in SICI, but by M1-intracortical circuits targeted by cTBS. Meanwhile, the spinal cord maintained the elevated level of excitability induced when expecting to observe movements, potentially useful to facilitate any required response to the movement observed.

## Introduction

It is well-established that the observation of movements executed by others induces changes in the observer's motor system (Fadiga et al., 1995). In this process the Mirror Neuron system (MNS) plays a pivotal role. The MNS was first described in monkey (Di Pellegrino et al., 1992) and at its heart contains neurons whose responses to an observed action are similar to the responses when the animal undertakes the same task (Gallese et al., 1996). It permits action understanding (Rizzolatti et al., 1996; Umilta et al., 2001) and has powerful influences on development (Rizzolatti and Craighero, 2004).

The original work in macaque described a system coding observation of actions upon objects (Di Pellegrino et al., 1992). Later it was shown that the system operates in human even during intransitive movements (Iacoboni et al., 1999). This has extended the potential for the human MNS to be involved in a much wider range of activities (Rizzolatti et al., 2009).

However, it is not clearly understood how the MNS is involved in the suppression of unwanted actions (Kraskov et al., 2009), since motor areas (including the M1) are “primed” by action observation (Hari et al., 1998; Aziz-Zadeh et al., 2002). In monkey, inhibition might take place at a spinal level (Stamos et al., 2010), but it is unknown if such a mechanism works in human, as derived from the results of H-reflex studies (Baldissera et al., 2001; Patuzzo et al., 2003; Borroni et al., 2005; Borroni and Baldissera, 2008). A spinal mechanism for suppression of motor replication during movement observation (MO) would fit well with the reported increase in the excitability of the M1 in humans (Fadiga et al., 1995). In this scenario the excitability of spinal cord circuitry would compensate the increased excitability at supraspinal centers.

On the other hand, a cortical locus for canceling motor replication during MO cannot be ruled out. Sub-sets of human cortical neurons respond differently during MO and execution, and some of these neurons might account for replication-suppression during mirror-facilitation induced by observation of hand or lip movements (Gazzola and Keysers, 2009; Mukamel et al., 2010). For this process the increase of M1-intracortical inhibition might have a role (Murakami et al., 2011). A cortical (M1) locus for replica-suppression during hand MO has also been shown in monkey (Vigneswaran et al., 2013).

Therefore, it may be suggested that during MO there is a balance in the excitability of cortical circuits allowing mirror facilitating activity within M1 while at the same time retaining the descending drive to the spinal cord; this study is aimed at evaluating if such a balancing mechanism is present in humans.

Here we explore the excitability of different cortico-spinal circuits during the observation of simple finger movements, in a series of experiments involving 66 healthy human subjects. We initially characterized the time-course of cortico-spinal excitability (CSE) at different delays from movement-onset, its specificity on muscle by muscle profiles, and its dependence on the kinematics of the movement observed. For this purpose we used single-pulse transcranial magnetic stimulation (sTMS) over M1. Subsequently, we explored the contribution of spinal or cortical circuits to this effect, and we focussed at the delays of interest from movement-onset by means of electric cervicomedullary stimulation (CMS) (Ugawa et al., 1991) and different TMS protocols. Continuous theta-burst stimulation over M1 (cTBS-M1) selectively suppresses the I_1_-wave circuits (Di Lazzaro et al., 2005); therefore this cortical circuit was evaluated by sTMS responses during MO before and after cTBS-M1. Measurements of short intra-cortical inhibition (SICI; later I-waves) and intra-cortical facilitation (ICF) were used to evaluate the effect of MO on the excitability of cortical circuits (Kujirai et al., 1993; Di Lazzaro et al., 1998).

We predicted that an increase of the excitability of cortical circuits during MO would be a marker for the mirror activity. However, cortical output should be suppressed in order to avoid unwanted motor replication. At the same time, the increased excitability at the spinal cord, resulting from expectancy prior to MO, would be maintained; perhaps useful to facilitate motor responses to the movement observed, if required (Arias et al., 2014).

## Materials and methods

All experiments presented here were approved by the University of A Coruña Ethics Committee. Subject's consent was obtained according to the Declaration of Helsinki.

### Experiments 1 and 2

Nineteen healthy volunteers participated in Experiment 1, where a movement lasting 200 ms was presented [8 male, average age 28 ± 1.7 years, 18/19 right-handed (Oldfield, 1971), all naïve to the protocol].

Seventeen healthy volunteers were recruited for Experiment 2, where a movement lasting 720 ms was shown [9 male, average age 27.8 ± 2.2 years, 16/17 right-handed (Oldfield, 1971), all naïve to the protocol].

**Figure 1:**
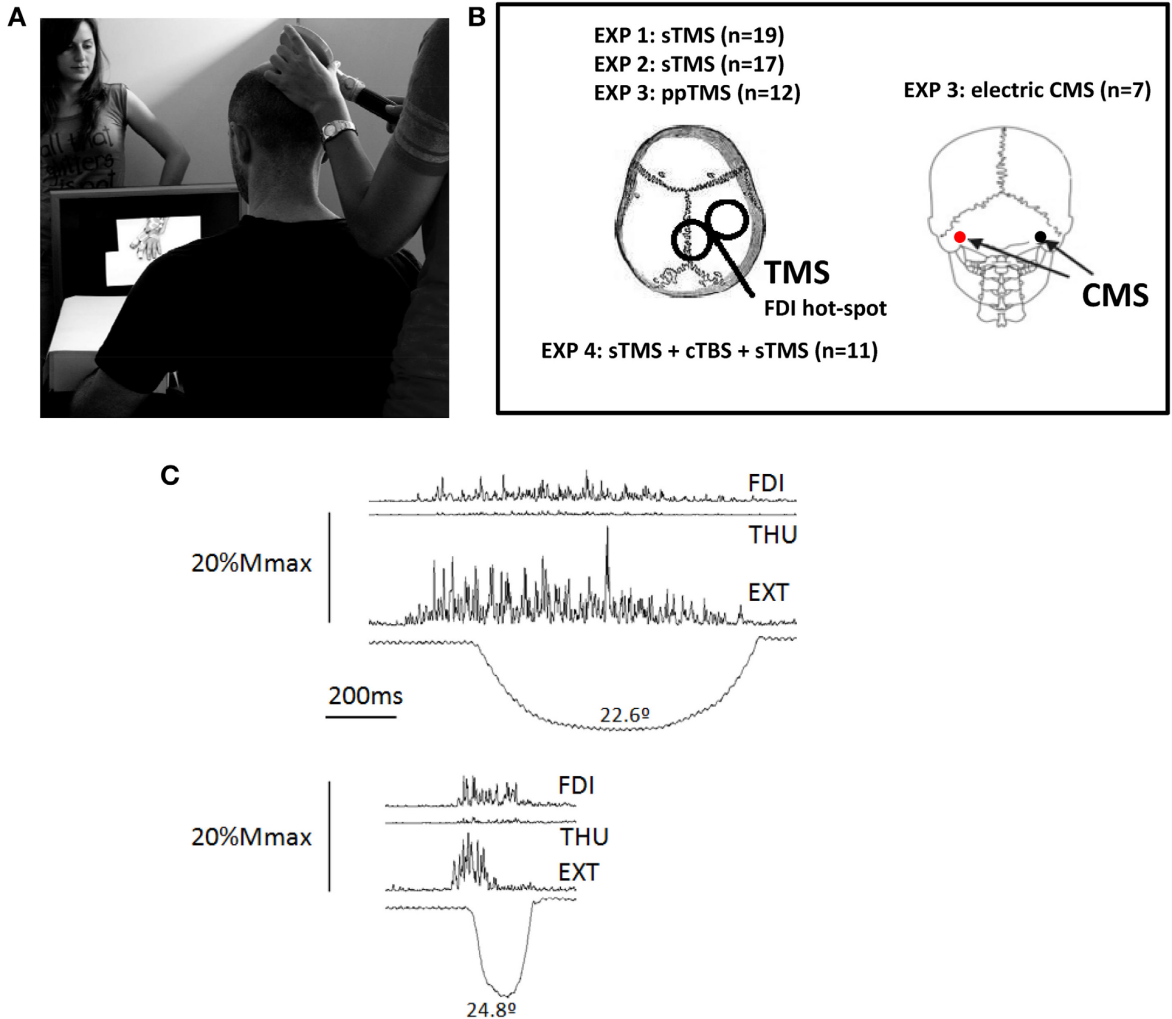
**(A)** Experimental Set-up. The person shown standing behind the screen was absent in the experimental sessions, she is shown here to illustrate the scale of the hand displayed on the screen. **(B)** Representation of the stimulations areas in the different experiments (EXP). TMS was applied using a figure of 8 coil. The coil was in all cases placed on the M1-representation of the first dorsal interosseus (FDI) muscle, on the right-hemisphere in all subjects. Electric cervico-medullary stimulation (CMS) was applied with the stimulating electrodes placed behind the mastoid processes; anode at the left, and the cathode at the right. **(C)** Activation of the three muscle explored in the experiments during a real index extension. One of the experimental subjects performed index finger extensions in a separate session, and several repetitions were recorded to produce finger extensions similar to that displayed in the video, (i.e., duration, symmetry, and peak amplitude). The EMG recordings were synchronized to the angular displacement of the index finger metacarpophalangeal joint, and their amplitude was scaled on the vertical, representing 20% of the amplitude potential of each muscle in response to supramaximal (M_*max*_) electric stimulation delivered at the Erb's point. The larger involvement of the main agonist, the extensor digitorum (EXT) is clear, but also involvement of the FDI and, to a much lesser extent, of the thumb opponent (THU). Abbreviations: sTMS, single pulse transcranial magnetic stimulation; ppTMS, paired-pulse TMS; cTBS, continuous theta burst stimulation.

All subjects had normal or corrected-to-normal vision and were medication-free in the week prior to the session. Subjects were seated comfortably on a chair with their left forearm resting on a table and both hands covered from view, while observing a PC monitor 1 m in front of them, on which was displayed a third's person left hand (Figure 1A). In each session comprising 4 sets, we presented 136 repetitions of the same left index-finger movement for observation. Repetitions were separated each other by a transitional Blank lasting 1–2 s. Twenty repetitions of the same hand not moving (“*Still*”) were also presented (in a ratio of 1:7 of the other stimuli), and in such cases the absence of movement was cued by the word “*Still*” in the preceding transitional Blank. We also included 4 *Stills* which were un-cued. Subjects were requested to notify the assistant when no movement was observed after the black-screen if not warned as “*Still*”; if the subject did not report the un-cued *Still*, data from the whole set was discarded as sign of non-attentiveness, and repeated (this occurred for 7 subjects, but only in 1 set). Un-cued *Stills* were present in Experiments 1 and 2, with the cued “*Still*” only in Experiment 1.

**Figure 2:**
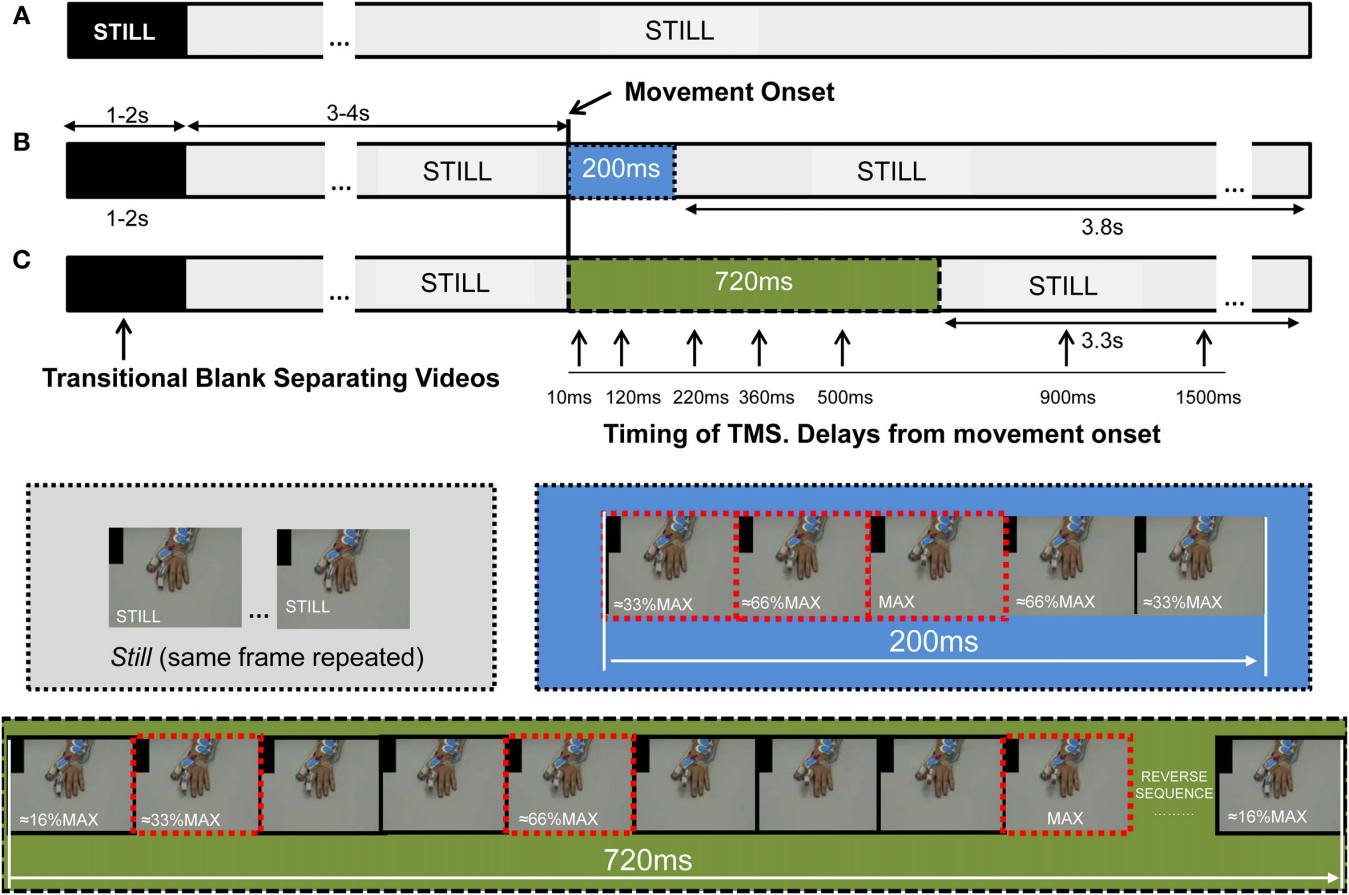
**The stimuli presented to the subject comprised several repetitions of video stimuli, each 8 s in duration**. Each video showed a single finger extension, lasting 200 or 720 ms, a hand remaining Still during the whole video (*Still*), or in some experiments, a Black video. **(A)** The *Still* stimulus was constructed from 8 s repetitions of the same frame; when displayed, the prior transitional Blank (of 1–2 s duration) overlapping the edge of the videos cued the presence of stillness with the word “*Still*.” **(B,C)** For the construction of the movement stimulus we used several frames showing the finger at different positions during the extension, and the reversal of these same frames allowed presentation of a retraction phase, such that it was of identical trajectory and angular velocity (blue and green sections for 200 and 720 ms). The 720 and 200 ms movements were embedded into the same Still video, but without cueing for Still. For the 720 ms video the frames showed the finger at about 16%, 33%…66%…and 100% of the maximal amplitude recorded (MAX, within the frame-images), and the reverse. The same frames were used to construct the 200 ms stimulus, but just using them in the sequence 33%, 66%, 100%, 66%, 33% of MAX (frames with dashed edges). This was done so that the movement started 3–4 s after hand appearance, subsequent to the Blank transition. Stimulation of the cortico-spinal tract was applied at specific delays from movement-onset (represented by vertical arrows) and at the same time for the Still video; its timing was controlled by the addition of a black spot in the upper left corner of the screen, which was covered from subject's view to avoid prediction. The black spot turned to white 80 ms prior to movement-onset, the change in color was captured with a photocell which trigger the interface controlling the stimulators and the recording systems. Abbreviations: TMS, transcranial magnetic stimulation.

For each repetition one TMS pulse was delivered, its timing relative to the observed movement was systematically but randomly explored (see below). The whole range of stimuli was presented in 4 sets, with 3 min rest between sets. The stimuli were prepared/assembled using Pinnacle System Software: The hand was filmed, and a single slow finger movement recorded. From the 25 fps recording, several frames were selected: 1 Still frame (the last frame preceding finger movement during recording) and 9 Movement frames at different finger positions. To create the Movement video stimulus, frames were arranged in this order: 3–4 s × Still frame + Movement frames + 3.3–3.8 s Still frame (Figure 2). To create the Still video all frames were repetitions of the Still frame (Figure 2A). The same frames were use to create the 200 ms (Figure 2B) and 720 ms (Figure 2C) movement. While the 720 ms movement frames sequence included 9 frames showing different finger positions during the extension phase recorded (≈16% MAX, ≈33% MAX,…. ≈66% MAX,… 100%MAX) and then reversed to show a symmetrical movement; the 200 ms 5 movement frame sequence was (≈33% MAX, ≈66% MAX, 100%MAX, ≈66% MAX, ≈33% MAX) (Figure 2); thus both movements were identical in amplitude and symmetry, but of different duration.

TMS-pulses were delivered using a monophasic Magstim 200^2^ stimulator (Magstim Company, Whiteland, Dyfed, UK) with 70 mm figure-of-8 coil. The coil was oriented tangentially to the skull, with the handle pointing 45° back/downwards to induce currents in the postero-anterior direction (Figure 1B). The stimulation intensity was 130% of individual rest motor threshold (RMT), calculated on the hot-spot of the first dorsal interosseous (FDI) on the right hemisphere, which was marked. RMT was the minimum intensity required to produce 50% of responses of ~50 μV in 10 consecutive trials (Rossini et al., 1994), while looking at a black screen and with the muscle at rest. Stimulation whilst watching the movies was applied over the hot-spot of the FDI on the right hemisphere, one pulse for each video stimulus. Timing of TMS-pulses relative to the observed movement was controlled by the addition of a 2 × 5 cm black rectangle in the Still frames (Figure 2), turning to white in the frame appearing 80 ms prior to movement-onset, and at the same time in the Still video stimuli. This region was obscured on the monitor observed by the participants but was covered by a photocell attached to the PC screen and sent to the CED1401 as the “video-trigger.” TMS was delivered at the following delays relative to the movement-onset: 10, 120, 220, 360, 500, 900, and 1500 ms (Figure 2). Delays were randomized across the experiment, (Signal Software; CED, UK) and the same intervals were used for control Still video stimuli.

#### Justification of delay intervals

The Control condition for possible changes in CSE during action observation was the 10 ms delay (Control), which permits the evaluation of CSE during the observation of a still hand while controlling the levels of MO expectancy present during the observation of movements (Arias et al., 2014). The 10 ms delay is not long enough for any significant visual processing to be induced by movement (Maunsell et al., 1999). One hundred and twenty milliseconds approximately corresponds to the time of maximum amplitude of the observed 200 ms-movement, and is within the time frame of visual information reaching the fast central movement regions [e.g., cortical area MT area (Schmolesky et al., 1998) in macaque]. Two hundred and twenty milliseconds was selected as neurons responding during observation and execution show peak excitation at ~200 ms (Mukamel et al., 2010); similarly increases in CSE induced by sequence of images of the hand occur at this time (Catmur et al., 2011). Three hundred and sixty and five hundred milliseconds were selected as they span the range of times where execution/observation responsive neurons show peak inhibition (Mukamel et al., 2010; Vigneswaran et al., 2013), 360 and 500 ms are at the maximum extension phase and the middle of the retraction phase of the 720 ms movement, respectively, 900 and 1500 ms were selected to investigate the dynamics of excitability after movement (Mukamel et al., 2010).

#### Data collection

Using Ag/AgCl surface electrodes via D360 amplifiers (Digitimer, UK), motor evoked potentials (MEP's, filtered between 3 and 3000 Hz) were recorded over three muscles involved in a finger extension (Darling and Cole, 1990) (Figure 1C), the FDI, thumb opponent (THU) and extensor digitorum (EXT) muscles. Signals were sampled at 10 kHz and stored via the CED1401. MEP amplitude and the level of background EMG (Thompson et al., 1991) (area/time from −80 to −10 ms relative to the TMS-pulse) were calculated with customized MatLab programmes (The Mathworks, USA). This procedure was repeated in Experiments 3 and 4.

#### Instructions given to the subjects

Subjects were required to view passively, paying attention to the movies, fixating on a probe attached to the tip of observed index finger. They were also instructed to be relaxed but if they wished to blink to try to do so during black screens.

### Experiment 3

#### Paired-pulse TMS (ppTMS)-group

Twelve healthy volunteers participated (4 male and 8 female, age range 24–37 years, all right-handed (Oldfield, 1971), and all naïve to the protocol). Subjects had normal or corrected-to-normal vision and were medication-free in the week prior to testing. Each subject received 146 TMS pulses in 2 sets (96 for each 720 ms-MOVE, and 48 BLACK movies, and 2 un-cued STILL; two subjects missed the un-cued STILL in one set, which was repeated). TMS was delivered using two monophasic Magstim 200^2^ stimulators connected with a Bi-Stim module (Magstim Company, Whiteland, Dyfed, UK) and a 70 mm figure-of-8 coil. The coil was oriented tangentially to the skull, with the handle pointing 45° back/downwards to induce currents in the postero-anterior direction (Figure 1B). For the paired-pulse tests, a pair of TMS pulses was delivered such that the first (conditioning stimulus) was given in advance of the second (conditioned or test stimulus) by 2–3 ms to assess short intracortical inhibition (SICI), and by 10–15 ms to assess intracortical facilitation (ICF) (Kujirai et al., 1993; Ziemann et al., 1996).

Test stimuli were set at an intensity of 125% RMT. The conditioning stimuli were set at 90% of the AMT (active motor threshold) (Ziemann et al., 1996; Di Lazzaro et al., 1999). RMT and AMT were calculated over the hot-spot of the FDI on the right hemisphere, which was marked. RMT was defined as in Experiments 1 and 2. For AMT the muscle was slightly activated (~5–10% of a maximal voluntary contraction) and the liminal response required was about 200 μV (Rossini et al., 1994).

#### CMS-group

Seven healthy subjects participated (4 male and 3 female, age range 26–43 years, all right-handed (Oldfield, 1971), and all naïve to the protocol). Experimental procedures were the same as in ppTMS protocol, except that subjects received one CMS for each movie, 720 ms-MOVE, or BLACK. The number of movies was reduced to a single set of 31 (20 of Movement, 10 Blank; with 1 un-cued Still, which was always correctly identified by the 7 subjects). CMS was applied with a Digitimer D180 stimulator connected to a pair of Ag–AgCl electrodes. Electrodes were placed behind the mastoid processes with the anode on the left and the cathode on the right (Figure 1B). Stimulation intensity was increased progressively to the highest intensity acceptable to the subject, which had a MEP of the same latency as intensities evoking liminal responses, such that as the induced MEP amplitude increased, there was no latency shift. The mean intensity used in the experiments was 600 V (s.e.m. 33).

Several repetitions of Still-videos were also presented in these experimental sessions, and previously reported (Arias et al., 2014).

#### Delays explored by ppTMS and CMS

Exploration of ppTMS and CMS were focused at Control delay and 500 ms after movement-onset exclusively. This was imposed for ethical reasons to reduce the number of CMS pulses delivered to the subjects, and in the case of the ppTMS to perform the experimental session in about 45–60 min, including rest periods. Five hundred milliseconds was selected because the effect at this time was significantly greater than that produced at 360 ms, as shown in Experiments 1 and 2.

#### Instructions given to the subjects

Those were as before in Experiments 1 and 2. Since we included repetitions of Black videos to normalize responses across different techniques, a small gray fixation square was included and appeared during the transitional Blank preceding Black-videos. Subjects were asked to fixate and continue to look at this point after the fixation target was removed. This also served to announce the forthcoming Black video.

### Experiment 4

#### Effect cTBS on CSE depression during action observation

Eleven out of thirteen healthy volunteers successfully completed the session (5 male and 6 female, age range 24–37 years, all right-handed (Oldfield, 1971), and all naïve to the protocol). Subjects had normal or corrected-to-normal vision and were medication-free in the week prior to testing.

***Brain stimulation and stimuli presentation***. The protocol involved first evaluating of CSE by means of single TMS pulses during MO (sTMS-PRE), then a subsequent protocol of 40 s-cTBS on the same M1 region and, 1 min after, a re-evaluation of CSE using sTMS-POST during MO.

During PRE and POST the subjects observed 48 repetitions of the movement lasting 720 ms; 24 repetitions of a cued-*Still*; 24 repetitions of the BLACK condition; and 2-uncued-*Stills;* uncued-*Stills* were not correctly identified by 2 out of the 13 participants, and these were discarded from further analyses (i.e., *N* = 11). During the randomized presentation of the stimuli sTMS monophasic-pulses were delivered at Control and 500 ms delays, and also during BLACK conditions, as in previous Experiments 1 and 2 (Figure 1B). Stimulation was delivered at the 130%RMT of the FDI and recordings were acquired as before. Prior to PRE, and with the coil positioned on the FDI-hot-spot, we also determined the RMT for THU and EXT, so that the stimulation intensity used was equivalent to 122% (SEM 2.6) of the THU-RMT; and 130.3% (SEM 1.9) of the EXT-RMT.

The cTBS-pattern was set in Signal 4 software that controlled a CED1401 mkII feeding a Magstim Rapid stimulator. Pulses were delivered through a figure of 8-coil that was positioned as in the rest of the experiments, on the FDI-hot-spot previously determined for PRE and POST testing. Before to cTBS, we determined the AMT of the three muscles, always on the same hot-spot as before, but now with the biphasic pulse (Magstim Rapid). cTBS was delivered at the 80% AMT-FDI (Di Lazzaro et al., 2005; Huang et al., 2005), which corresponded to a 76.3% (SEM 2.2) of the THU-AMT; and a 78.6% (SEM 2.9) of the EXT-AMT, none of the subjects reported adverse effects due to cTBS.

### Statistical analysis

The SPSS (SPSS Inc) software version 15.0 was used to analyze the data. The analyzed variables were the same for all experiments:

- MEP amplitude: mV (peak-to-peak of the MEP).
- EMG-background: mV (mV^*^ms normalized in time).

Normality of the distributions was checked by Kolgómorov-Smirnov tests for one sample. Univariate analyses of variance with repeated measures (ANOVA_RM_) were used in all experiments. It evaluated changes as a function of the observed action and time-course from movement onset. The Mauchly test was used to evaluate sphericity; if violated, the Greenhouse coefficients (Ԑ) were employed to correct the degrees of freedom. The Bonferroni's correction was applied for multiple pair-wise comparisons. Significance set at *p* ≤ 0.05.

*Experiment 1* specifically evaluated the modulation of the CSE at different delays. We used an ANOVA_*RM*_ with two within subject factors: DELAY had 7 levels (Control, 120 ms… 1500); and MUSCLE with 3 levels (FDI, THU, and EXT).

The analysis of the CSE rebound-effect included a condition not dependent on delay from movement-onset. It was evaluated by an independent ANOVA_RM_, with factor CONDITION (500 ms, Still, and 1500 ms) and factor MUSCLE (as before).

Before conducting the analysis we normalized the data. Then, for each muscle, we calculated the mean value at the Control delay across all subjects, which served as the divisor for the rest of the delays for that give muscle.

Subsequently, *Experiment 2* included a group of subjects observing movements lasting 720 ms. We normalized the data (as before, average of Control responses) and analyzed the difference in modulation of CSE in the two groups, inspecting DELAY and MUSCLE and a between-subjects factor GROUP (levels G200, G720).

For *Experiment 3* the ANOVA_RM_ was similar to Experiment 2 (DELAY × MUSCLE × GROUP), but DELAY included only Control and 500 ms delays, and GROUP the TMS-group and CMS-group. The amplitudes of the MEPs were normalized (TMS and CMS are not directly comparable). For each muscle and group we calculated the average of the response in the Black condition, which became the divisor for the Control and 500 ms delays in the corresponding muscle and group.

In *Experiment 4*, we analyzed the responses at Control and 500 ms delays in the three muscles, and the effect of cTBS. The ANOVA included DELAY, MUSCLE, and TIME-POINT evaluation (*before* and *after* cTBS). We also statistically evaluated the effect of cTBS on Black and Still responses in the same way, but excluding the factor DELAY. Before conducting the ANOVA, data was normalized; for each muscle we calculated the average of all subjects' responses *before* and *after* cTBS in the Black condition, which served as divisor for all values in the corresponding muscle.

## Results

### Experiment 1

In the first experiment subjects passively observed repetitions of the movement lasting 200 ms. Interleaved at random between the sequences with movements they also observed trials showing the same hand but not moving (*Still*), which was cued in advance of the presentation. The TMS pulses were delivered at specific delays from movement-onset, and the *Control* delay was delivered before MO but when it was also expected to be observed. The *Still* repetitions served to evaluate any rebound effect on CSE modulation during MO.

**Figure 3:**
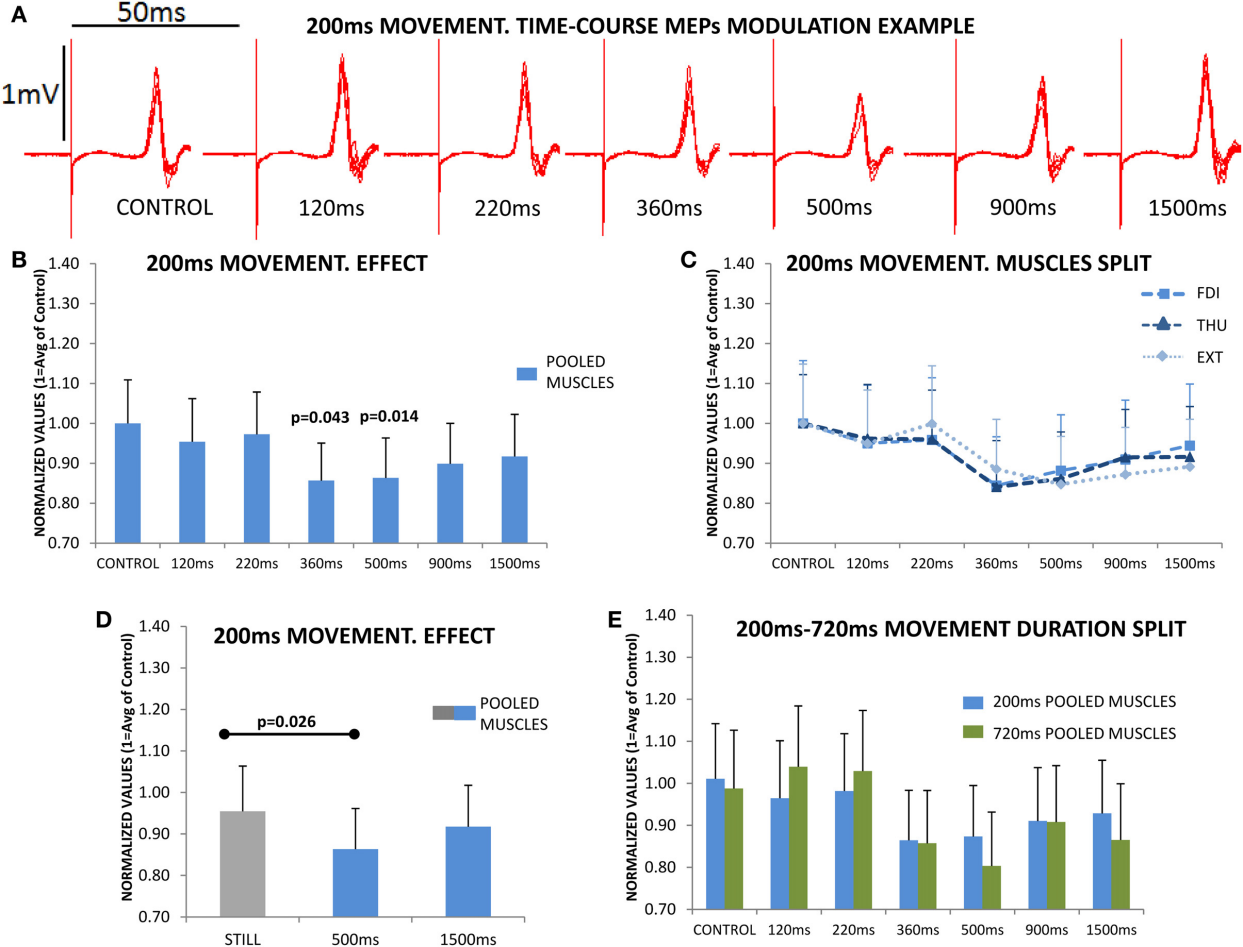
The upper section **(A)** shows the overlaid traces from a representative subject at the different delays, and acquired during the observation of one of the 4 sets of the 200 ms movements. The reduction in the peak-to-peak amplitude of the motor evoked potential (MEP) immediately following the stimulus artifact at the 500 ms delay is obvious. **(B)** shows the mean subjects (*n* = 19) responses. MEP amplitudes were significantly reduced at 360 and 500 ms, and then recovered; the figure represents the responses of the three muscles pooled since they behaved similarly during movement observation (no significant differences); this can be observed in section **(C)** where the time-course modulation of the three muscles overlapped. **(D)** plots the responses during Still, which was significantly different compared to the 500 ms response, but not compared to 1500 ms response, again in all muscles. **(E)** shows the time-course modulation during the observation of movements lasting 200 ms (*N* = 19) and 720 ms (*N* = 17); normalized MEP amplitudes at each of different delays were never significantly different for 200 and 720 ms. Values are the mean and 1 s.e.m.

Figure 3A shows overlaid traces of the CSE exploration at the different delays in a representative subject. CSE was significantly modified across the different delays during MO [*F*_(6, 108)_ = 5.860; *p* < 0.001]. Figure 3B shows this pooled-muscle effect, which was not significantly different for the three muscles evaluated [*F*_(12, 216)_ = 0.966_ε=0.428_; *p* = 0.444]. This is shown in Figure 3C which illustrates the overlapping of the responses of the three muscles. The three muscles showed a significantly reduced CSE (reduced MEPs) at 360 and 500 ms after movement-onset (*Bonferroni p* = 0.043 and *p* = 0.014, Figure 3B).

Subsequently we asked if the effect in the three muscles was larger than that obtained during the *Still* condition, which therefore had no effect due to MO expectancy. For this, we compared the magnitude of the effect at 500 ms and at the longer delay of 1500 ms, vs. the cued-*Still* hand, showing that there was a significant difference [*F*_(2, 36)_ = 4.338; *p* = 0.021]. *Post-hoc* testing indicated a significantly reduced CSE at 500 ms compared to *Still* (*Bonferroni p* = 0.026), while at 1500 ms the CSE had recovered to similar levels to *Still* (*Bonferroni p* = 0.887), Figure 3D.

These results show an active reduction of the CSE in the three muscles examined during MO, which is more than can be explained by the mere removal of MO expectancy, and is present for observation of a movement lasting 200 ms.

### Experiment 2

In the second experiment we asked if this effect was dependent on the duration of the movement observed. For this, we compared the modulation of the CSE at the different delays during the observation of a 200 ms movement, to the profile resulting from observing a movement of the same amplitude and symmetry (extension and retraction), but of 720 ms duration. Again, the CSE was significantly modulated at specific delays during MO [*F*_(6, 204)_ = 12.278_ε=0.647_; *p* < 0.001] and the effect was not differentially expressed in the three muscles [F_(12, 408)_ = 1.319_ε=0.496_; *p* = 0.251]; remarkably these effects were not significantly different for the groups observing a movement lasting 200 or 720 ms [*F*_(12, 408)_ = 0.855_ε=0.496_; *p* = 0.529]. This can be observed in Figure 3E, which shows 200 vs. 720 ms responses at each delay.

### Experiment 3

While Experiments 1 and 2 explain a reduction of the CSE during the observation of movements, the TMS technique does not allow us to localize the origin of such an effect. In Experiment 3 we asked if this effect could be explained by the modulation of the spinal cord circuits, for which we used electric CMS; or by the modulation of intracortical circuits, explored by paired-pulse TMS. During these experiments subjects observed repetitions of the movement lasting 720 ms, and the stimulation was delivered at the Control and 500 ms delays. We also included several randomized videos showing a Blank (Black), which served to normalize data and allow the comparison of the different techniques (TMS and CMS).

**Figure 4:**
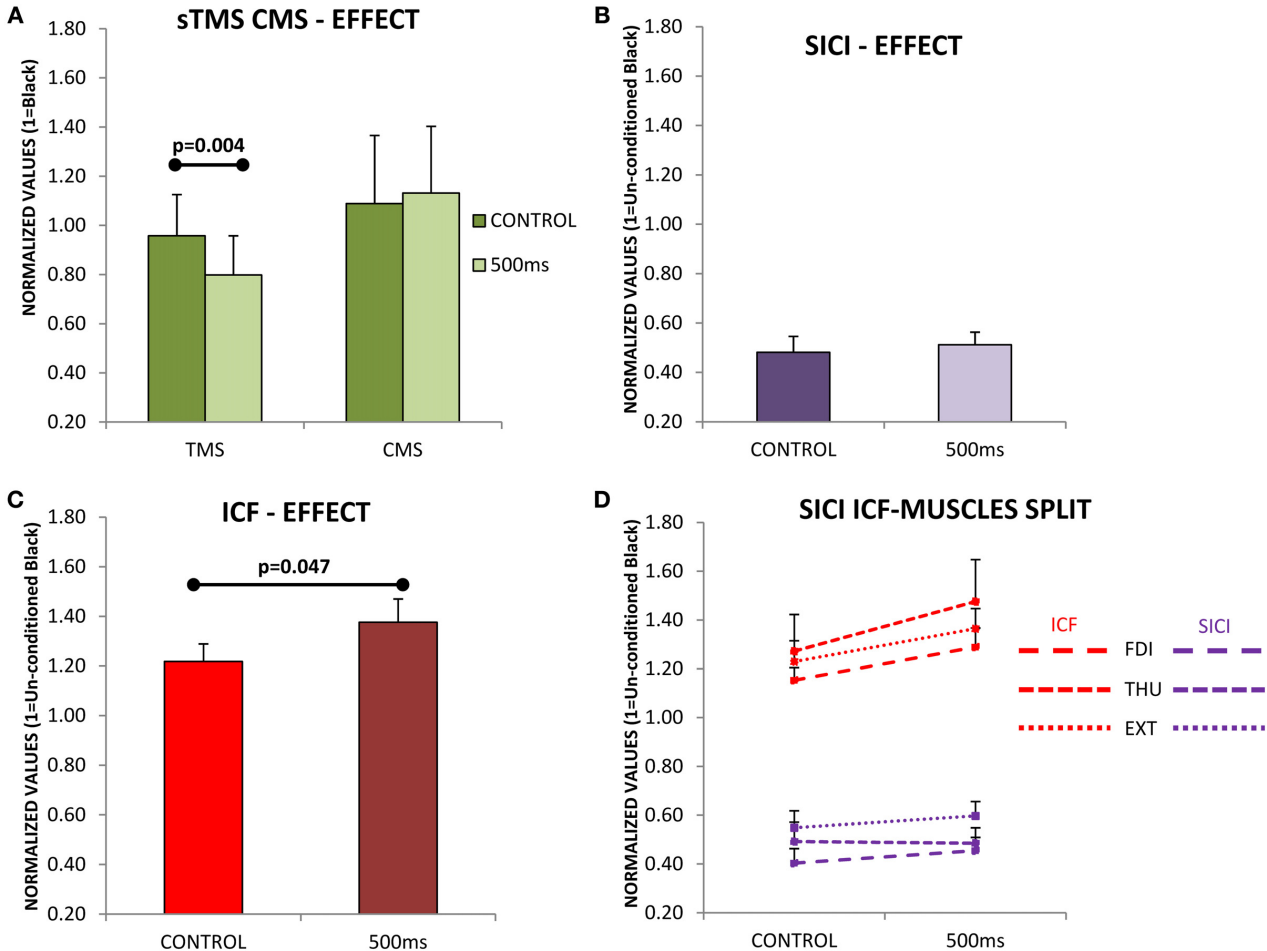
**(A)** Evaluation of cortico-spinal excitability (CSE) and spinal excitability (SE) during the observation of movements lasting 720 ms. Subjects were evaluated at two different time points (Control and 500 ms) with single pulse transcranial magnetic stimulation (TMS; *N* = 12) in the case of CSE, and with cervico-medullary stimulation (CMS; *N* = 7) for SE. Only significant differences were found between Control and 500 ms when TMS was used. **(B)** Short-intracortical-inhibition (SICI) responses at the Control and 500 ms delays, SICI was not influence by movement observation. **(C)** Intracortical-facilitation (ICF) was significantly increased at 500 ms after movement-onset. **(D)** ICF and SICI separately in each muscle (FDI, first dorsal interosseous; THU, thumb-opponent; and EXT, extensor digitorum). No significant differences were found between muscles. Values are the mean and 1 s.e.m.

Figure 4A shows that the CSE evaluated with single pulse TMS is significantly reduced at 500 ms after movement-onset [*F*_(1, 11)_ = 12.955; *p* = 0.004], and this effect was not differentially observed in the three muscles [*F*_(2, 22)_ = 0.209; *p* = 0.813]. It is clear from Figure 4A that the modulation of the SE (using CMS) was significantly different to that observed with TMS [*F*_(1, 17)_ = 6.324; *p* = 0.022]; also the modulation of the SE was not significantly different at 500 ms compared to Control [*F*_(1, 6)_ = 0.342; *p* = 0.580, Figure 4A], and this was not differentially expressed for the three muscles [*F*_(2, 12)_ = 0.006; *p* = 0.992]. These results therefore exclude a role of SE in the reduction of CSE observed at 500 ms during MO.

Subsequently, we asked if an increase in SICI at 500 ms could be responsible for such an effect, but SICI was not significantly modified at 500 ms compared to Control [*F*_(1, 11)_ = 0.925; *p* = 0.357, Figure 4B]. Finally, instead of a reduction of the ICF at 500 ms compared to Control, which might explain the drop in CSE, the circuits explored showed a significant increase in excitability [*F*_(1, 11)_ = 5.004; *p* = 0.047, Figure 4C]. The effects seen were not significantly different in the three muscles under study, both for SICI [*F*_(2, 22)_ = 1.215; *p* = 0.316, Figure 4D purple traces], or ICF [*F*_(2, 22)_ = 0.309_ε=0.667_; *p* = 0.651, Figure 4D red traces].

### Experiment 4

Thus, far we have observed a significant reduction of the CSE at 500 ms during MO which is not explained by changes in the excitability of the spinal motorneurons. The effect was also not explained by a modulation of the excitability in the inhibitory circuits of M1, as explored by SICI (circuits generating I_2_–I_3_ descending activity). However, conversely, we observed an increase in cortical excitability of the circuits investigated by ICF. In Experiment 4 we explored the specific modulation of cortical circuits generating I_1_ descending volleys at the time of increasing ICF (500 ms after movement-onset), suggesting it to be responsible for the net decrease in CSE. The subjects observed the videos while single TMS pulses were delivered (PRE) at Control and 500 ms delays and, in addition, during Black and Still videos (all randomized in order). After this, 40 s of cTBS was applied to M1 to inhibit the intracortical circuits generating I_1_-descending activity, and single pulse TMS was delivered (POST), as before.

**Figure 5:**
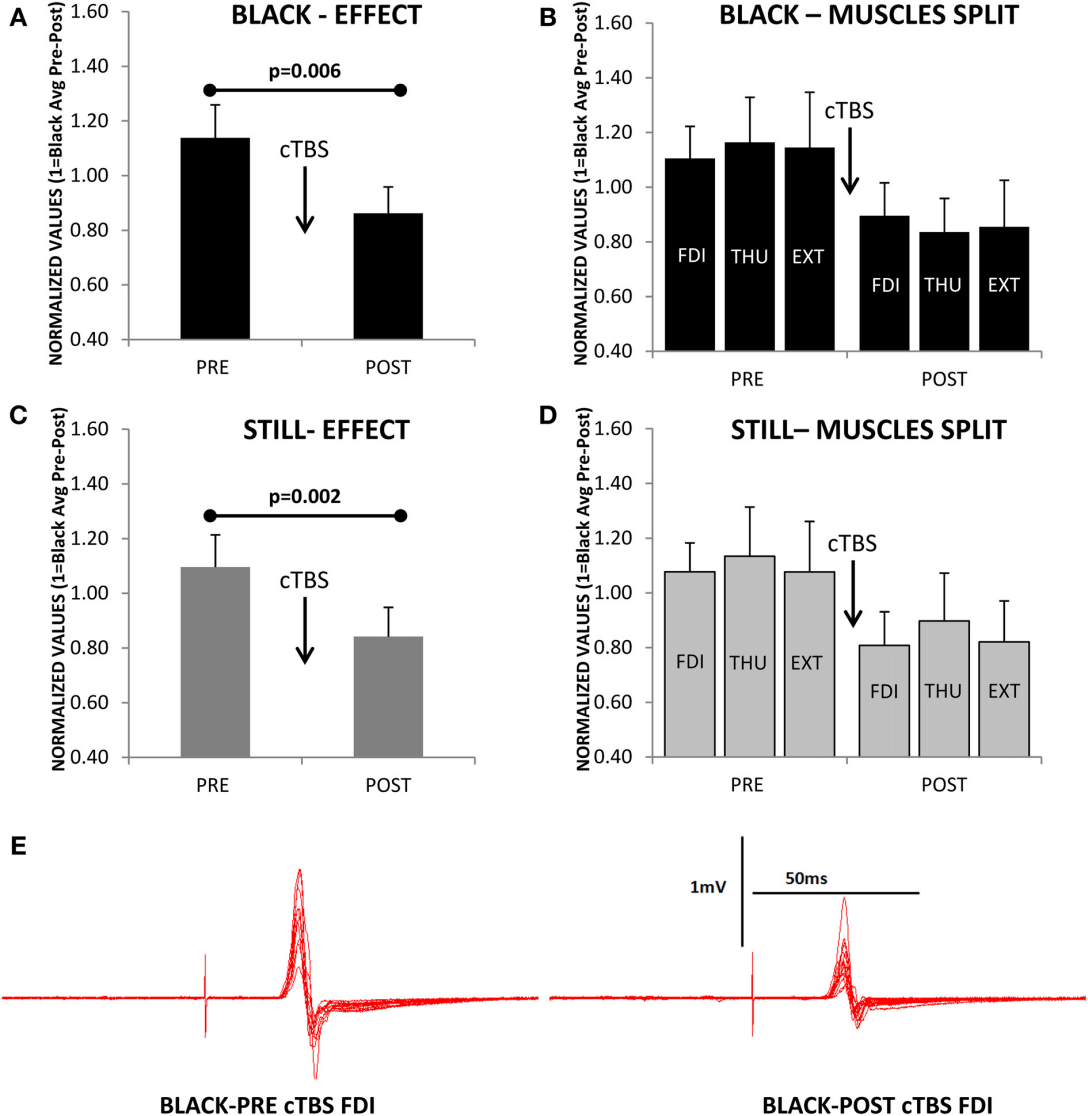
**(A)** Reduction of cortico-spinal excitability (normalized values of motor evoked potentials) as consequence of continuous theta-burst stimulation (cTBS) during the observation of the Black stimulus. **(B)** Same effect as in A but for each muscle separately (FDI, first dorsal interosseus; THU, thumb-opponent; and EXT, extensor digitorum). The three muscles were not differently modulated by cTBS. **(C,D)** A significant effect is also observed for the Still condition. **(E)** Representative example of motor evoked potentials decrease after cTBS recorded in the FDI while observing the Black stimulus. Values are the mean and 1 s.e.m., (*N* = 11).

Figure 5A shows a significant effect of cTBS on modulating cortical output [*F*_(1, 10)_ = 11.996; *p* = 0.006], expressed similarly in all three muscles [*F*_(2, 20)_ = 0.584; *p* = 0.567, Figure 5B], and obtained during the observation of the Blank. Remarkably, the CSE was also significantly reduced by the cTBS during the observation of a Still hand [*F*_(1, 10)_ = 17.331; *p* = 0.002, Figure 5C], and the three muscles did not show any significant different responses to this effect [*F*_(2, 20)_ = 0.061; *p* = 0.941, Figure 5D]. A representative example on the hot-spot, FDI, during Black is shown in Figure 5E.

**Figure 6:**
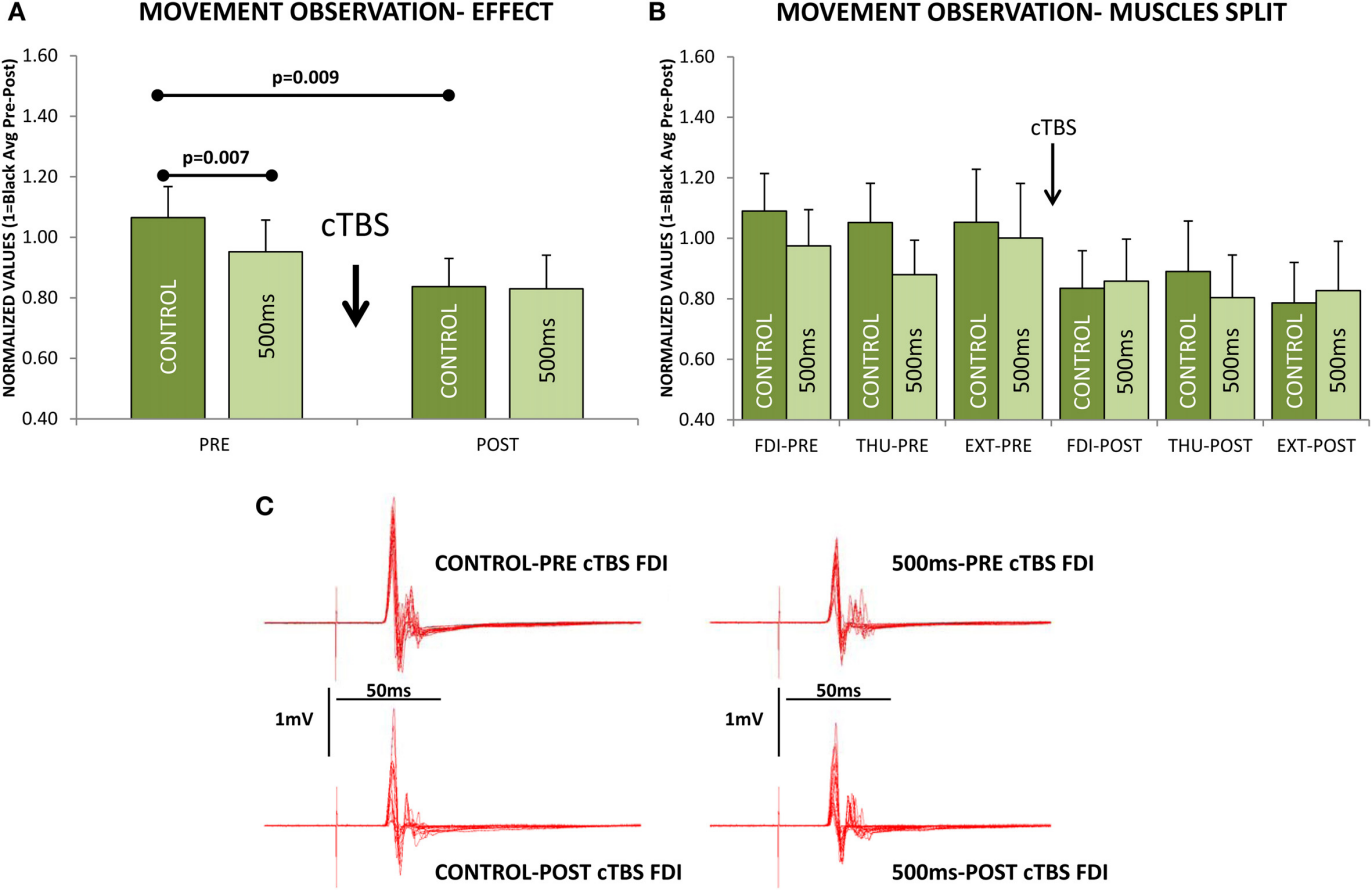
**Effect of continuous theta-burst stimulation (cTBS) during movement observation at the Control and 500 ms delays. (A)** Significant reduction in the cortico-spinal excitability (CSE) at 500 ms compared to Control obtained before cTBS (PRE). cTBS made the responses at the Control and 500 ms not significantly different at POST. The effect was due to a significant reduction of the CSE at the Control delay, as it differed significantly when comparing Control responses at PRE vs. POST. The cTBS did not reduce significantly the responses, comparing PRE vs. POST at the 500 ms delay. **(B)** Same effect as in **(A)** but for each muscle separately (FDI, first dorsal interosseus; THU, thumb-opponent; and EXT, extensor digitorum), muscle responses were not significantly different. **(C)** Representative traces recorded from one subject. Values are the mean and 1 s.e.m.

If we focus on the effects during MO at the two different delays from movement-onset, we also observed a significant modulation of the excitability induced by cTBS [*F*_(1, 10)_ = 5.982; *p* = 0.03] and remarkably, the effect was significantly different at Control and 500 ms delays [*F*_(1, 10)_ = 5.361; *p* = 0.043, Figure 6A]; but not seen in a significantly different manner for the three muscles [*F*_(2, 20)_ = 0.421; *p* = 0.662, Figure 6B]. Therefore, the CSE at PRE (before cTBS) was significantly different between Control and 500 ms (*Post-hoc test p* = 0.007) in agreement with Experiments 1–3, but not at POST, after cTBS (*Post-hoc p* = 0.873, Figure 6A). This effect is explained because the CSE at the Control delay was significantly reduced by cTBS (*Post-hoc* test *p* = 0.009), whereas at 500 ms the CSE was not significantly modified by cTBS (*Post-hoc* test *p* = 0.155, Figure 6A). Figure 6C shows a representative example (one subject) of the recordings obtained with stimulation at the hot-spot.

In each the sets of results reported above (Experiments 1–4) the MEPs were acquired in conditions where the levels of EMG background activity were not significantly different between delays and/or conditions.

## Discussion

We have evaluated a variety of cortical and spinal circuits during the observation of simple intransitive movements. The overall CSE was tested with single pulse TMS (Hallett, 2000). The excitability of the spinal-motorneurons was evaluated using CMS (Ugawa et al., 1991). We probed some specific circuits mediated by GABAergic M1-interneurons and responsible for the inhibitory control over cortico-spinal neurons, such as those generating the later I waves in response to ppTMS (SICI) (Di Lazzaro et al., 1998). Intracortical circuits producing I_1_ descending volleys were explored with single pulse TMS combined with cTBS (Di Lazzaro et al., 2005); ppTMS (ICF) was also used to understand the modulation of cortical facilitatory circuits (Kujirai et al., 1993) during MO.

We characterized an observable reduction of the CSE, significant at 360 and 500 ms after movement-onset, which afterwards recovered, and was present for observed movements of different angular velocities. Direct comparison of CSE modulation during each movement (200 and 720 ms) revealed that the CSE was not differentially modulated for the duration of the movement.

It is noteworthy that the process was not dependent on specific muscles or movement phase. The reduction of the CSE at 360 ms after movement onset was at the end of the up movement phase of the observed movement which lasted 720 ms, and remained at 500 ms after movement onset, already at the descending phase of the 720 ms-movement. It is also remarkable that 360 and 500 ms were time-points at which the movements had been completed when movement duration was 200 ms; however the reduction in CSE was also significant at those time points, and recovered only thereafter. We suggest it might reflect a stereotyped mechanism during MO, potentially triggered at movement onset.

Remarkably, the CSE suppression was appeared to be an active process, not explained by the mere removal of the effect of MO expectancy on CSE (Arias et al., 2014). Otherwise CSE at 500 ms would not be below the excitability level induced by the Still-hand, and this was a significant effect observed in our study.

Afterwards, we explored specific cortical and spinal circuits during MO, and focused at the delays of interest (500 ms and Control). Therefore, a different modulation of excitability might be present at different time points (Fadiga et al., 1995; Brighina et al., 2000; Strafella and Paus, 2000; Baldissera et al., 2001; Clark et al., 2004; Molnar-Szakacs et al., 2005; Montagna et al., 2005; Alaerts et al., 2009b; Koch et al., 2010; Catmur et al., 2011; Donne et al., 2011).

ICF, a marker of cortical excitability, was found to be elevated in our study, suggesting that some cortical circuits were boosted by MO at 500 ms after movement-onset, in agreement with data obtained in monkeys during execution and observation (Vigneswaran et al., 2013). Remarkably this effect occurred in parallel with an overall reduction in the CSE with a similar time-course. The most parsimonious explanation is that this is a way to allow mirror activity in M1 without overt motor replication, a finding which has already been described in monkeys (Vigneswaran et al., 2013).

The production of ICF by ppTMS is not well-defined by pharmacological studies (Di Lazzaro et al., 2003), and its dynamics might be dispersed and not bound to inter I-wave latency (Reis et al., 2008). There must be, in all cases, a way to counter the increment of cortical excitability, as determined by ICF, in order to reduce CSE (Kraskov et al., 2009; Vigneswaran et al., 2013), perhaps involving those circuits responsible for the I-wave (Murakami et al., 2011) or, perhaps, acting at the spinal cord level (Stamos et al., 2010).

However, the spinal cord excitability is increased by MO expectancy (Arias et al., 2014), and it remained stable at 500 ms after movement-onset in our study. An increase in SICI is reported in case of facial MO without replication (Murakami et al., 2011), but we did not observed any effect on SICI to compensate ICF while observing hand movements. This might be due to the sensitivity of different SICI protocols, but it is just possible that SICI differs while observing facial or hand movements. This possibility is supported by different functioning of cortical “mirror” neurons for facial and hand MO (Mukamel et al., 2010). Likewise, the fact that SICI did not increase during MO (as observed in our study) might be important in case an efficient response to the perceived movement is required (Reynolds and Ashby, 1999; Zoghi et al., 2003).

CSE modulation by MO, before and after cTBS, indicates that the cortical circuits generating I_1_ descending activity (un-explored by SICI, ICF, or CMS, but recruited by single pulse TMS; Ugawa et al., 1991; Di Lazzaro et al., 1998, 2006) are the candidates to compensate for the increase in ICF during MO. We observed that the significant reduction (or disfacilitation) in the CSE when we compared Control vs. 500 ms was absent after cTBS. Remarkably, this effect was due to a cTBS-induced reduction of the excitability at Control delay (as well as Black and Still), but not at 500 ms after movement-onset. We suggest that the reduction in the excitability observed at 500 ms in Experiments 1–3, and during Experiment 4-PRE, was linked to the cortical circuits generating I_1_ activity, the same circuits specifically modulated by cTBS (Di Lazzaro et al., 2005), and for this reason cTBS had no influence at 500 ms. Thus, the cancelation of the excitation detected in some pools of cortical neurons during MO in monkey and human (Mukamel et al., 2010; Vigneswaran et al., 2013) might take place at the same timing after movement-onset on specific (I_1_) circuits in the motor cortex.

The net process, described here for first time in human, is compatible with overt replication cancelation during the observation of hand actions (Kraskov et al., 2009; Vigneswaran et al., 2013) and with M1-facilitation by the MNS during MO (Fadiga et al., 1995). At the same time the spinal cord remains ready (Mellah et al., 1990; Arias et al., 2014) in case the observer requires to make a response. From a mechanist point of view, the lack of modulation of SICI-circuitry is convenient to facilitate the whole process, and to guarantee the availability of a potential motor response (Reynolds and Ashby, 1999; Zoghi et al., 2003).

### Task observed and perspective taken

The process we describe is triggered by a very simple intransitive movement during plain observation. No motor act was made or planned (Gangitano et al., 2001, 2004) as this is known to modulate the activity of the motor system (Decety et al., 1997). However, plain observation with no movement performance might involve a motor plan to stay still. In our experiments subjects were told to avoid any movement at the time of observation. This “conscious suppression” could have had an influence on the results, given that the mirror neuron network is involved in the inhibition or release (regulation) of intentional or automatic imitation (Brass et al., 2005; Bien et al., 2009).

Observation of movements of different complexity might also influence the modulation of the CSE of the observer. Thus, complex or/and forceful movements are known to induce greater facilitation of M1 (Alaerts et al., 2010), also affecting spinal excitability (Baldissera et al., 2001; Patuzzo et al., 2003; Borroni et al., 2005, 2008; Montagna et al., 2005; Borroni and Baldissera, 2008), which might override the stereotyped inhibition described herein.

It also is conceivable that the effect we observed at 500 ms starts earlier (present at 360 ms also for inhibition in our study). In line with this idea is the fact that mirror facilitation is observed about 200 ms after onset of simple intransitive movements, if MNS structures have been primed by observation in the 1st person perspective (Lepage et al., 2010; Catmur et al., 2011). Thus, perspective might differentially modulate the excitability of the motor system during MO (Maeda et al., 2002; Schutz-Bosbach et al., 2006; Alaerts et al., 2009a).

### Concluding remarks

Our results support different roles for several cortical circuits during MO. Critically, it is known that the application of TMS recruits different cortico-spinal circuits depending on pulse intensities, direction of the induced currents, pulse-wave forms or coil type (Di Lazzaro et al., 2008), and the effect of stimulation parameters on the modulation of CSE during MO is known (Loporto et al., 2013). This might explain differences between published studies.

In conclusion, during human MO there is a phase of stereotyped modulation in the excitability of cortico-cortical circuits. This permits an early MNS facilitation in M1, but which is suppressed also at cortical level, perhaps aiming at avoiding overt motor replication. At the same time, the spinal circuits maintain the level of excitability induced by MO expectancy, which might be boosted if the observer wishes or requires respond to the action.

## Author contributions

Conception and design of the work: Pablo Arias, Verónica Robles-García, Antonio Oliviero, Javier Cudeiro. Acquisiton: Pablo Arias, Verónica Robles-García, Yoanna Corral-Bergantiños, Laura Mordillo-Mateos, Kenneth Grieve, Antonio Oliviero, Javier Cudeiro. Analysis: Pablo Arias, Verónica Robles-García, Nelson Espinosa, Antonio Oliviero, Javier Cudeiro. Interpretarion: Pablo Arias, Verónica Robles-García, Yoanna Corral-Bergantiños, Nelson Espinosa, Laura Mordillo-Mateos, Kenneth Grieve, Antonio Oliviero, Javier Cudeiro. Drafting the work: Pablo Arias, Kenneth Grieve, Javier Cudeiro. Critical revision: Pablo Arias, Verónica Robles-García, Yoanna Corral-Bergantiños, Nelson Espinosa, Laura Mordillo-Mateos, Kenneth Grieve, Antonio Oliviero, Javier Cudeiro. Final approval: Pablo Arias, Verónica Robles-García, Yoanna Corral-Bergantiños, Nelson Espinosa, Laura Mordillo-Mateos, Kenneth Grieve, Antonio Oliviero, Javier Cudeiro.

### Conflict of interest statement

The authors declare that the research was conducted in the absence of any commercial or financial relationships that could be construed as a potential conflict of interest.
